# Chitosan-modified hollow manganese dioxide nanoparticles loaded with resveratrol for the treatment of spinal cord injury

**DOI:** 10.1080/10717544.2022.2104957

**Published:** 2022-07-28

**Authors:** Yingqiao Li, Zhiru Zou, Jinyu An, Qian Wu, Le Tong, Xifan Mei, He Tian, Chao Wu

**Affiliations:** a Pharmacy School, Jinzhou Medical University, Jinzhou, Liaoning, China; b Department of Orthopedics, Third Affiliated Hospital of Jinzhou Medical University, Jinzhou, Liaoning, China; c Key Laboratory of Medical Tissue Engineering of Liaoning Province, Jinzhou Medical University, Jinzhou, Liaoning, China; d Department of Histology and Embryology, Jinzhou Medical University, Jinzhou, Liaoning, China

**Keywords:** Oxidative stress, inflammation, blood–spinal cord barrier, apoptosis

## Abstract

Spinal cord injury (SCI) is a serious central nervous system disease, and secondary injury, including oxidative stress, the inflammatory response and accompanying neuronal apoptosis, will aggravate the condition. Due to the existence of the blood–spinal cord barrier (BSCB), the existing drugs for SCI treatment are difficulty to reach the injury site and thus their efficacy is limited. In this study, we designed chitosan-modified hollow manganese dioxide nanoparticles (CM) for the delivery of resveratrol to help it pass through the BSCB. Resveratrol (Res), a poorly soluble drug, was adsorbed into CM with a particle size of approximately 130 nm via the adsorption method, and the drug loading reached 21.39 ± 2.53%. *In vitro* dissolution experiment, the Res release of the loaded sample (CMR) showed slowly release behavior and reached about 87% at 36 h. *In vitro* at the cellular level and *in vivo* at the animal level experiments demonstrated that CMR could alleviate significantly oxidative stress by reducing level of reactive oxygen species (ROS), malondialdehyde (MDA), superoxide dismutase (SOD), and increasing glutathione peroxidase (GSH) level. Additionally, immunofluorescence (iNOS, IL-1β, and Cl caspase-3) and western blot (iNOS, cox-2, IL-1β, IL-10, Cl caspase-3, bax, and bcl-2) were used to detect the expression of related factors, which verified that CMR could also reduce inflammation and neuronal apoptosis. These results indicated that CM, as a potential central nervous system drug delivery material, was suitable for SCI treatment.

## 1. Introduction

Spinal cord injury (SCI) is a common central nervous system injury that can lead to spinal cord nerve conduction dysfunction with symptoms such as limb pain, paralysis, urinary disorders, and even life-threatening, causing huge mental and economic burdens to the patient’s individual, family, and society (Hall et al., 2019; Kobashi et al., 2020). The SCI course includes primary injury and secondary injury (Liu & Xu, 2010). Primary injury refers to injury caused by an external force directly or indirectly acting on the spinal cord, which is an irreversible process (Rowland et al., 2008). Secondary injury is a pathological change that gradually develops on the basis of primary injury, and includes oxidative stress, inflammatory responses, ischemic cell apoptosis, and bleeding, which further exacerbate the injury and seriously hinder the recovery of the disease (Ahuja et al., 2017). Oxidative stress and the inflammatory response are especially important factors in SCI progression (Silva et al., 2014). Therefore, inhibiting oxidative stress and the inflammatory response after SCI has become a recognized method for the treatment of SCI.

In recent years, with the rapid development of nanomedicine, nanomaterials have attracted more attention in the treatment of SCI, and include polylactic acid-glycolic acid copolymer (PLGA) nanoparticles, cerium oxide nanoparticles, chitosan nanoparticles, and manganese oxide (MnO_2_) nanoparticles (Caron et al., 2014; Li et al., 2019; Rizeq et al., 2019; Lord et al., 2021). These carrier materials have the common characteristics of small particle size, low toxicity, and biodegradability. Among them, MnO_2_ nanoparticles possess better potential as drug transporters due to their good catalytic effect in addition to the above characteristics. It can catalyze and decompose reactive oxygen species (ROS) into water and oxygen, thereby reducing the level of ROS and alleviating oxidative stress (Broughton et al., 1947; Kumar et al., 2019). Currently, the nanostructures of MnO_2_ include nanoparticles, nanosheets, and hollow manganese dioxide (HM) (Abbasi et al., 2015). Compared with MnO_2_ nanoparticles and nanosheets, HM has a large cavity structure, which enables it to carry a large number of insoluble drugs (Song et al., 2016; Yang et al., 2017). As a drug delivery carrier, it has been widely applied to diseases of the central nervous system, such as ischemic stroke and glioblastoma, which shows high scientific research value and clinical potential (Yang et al., 2021; Dong et al., 2022). The principle of improving the water solubility of insoluble drugs is the steric confinement of nanoscale hollow structures can reduce the crystallinity of poorly soluble drugs, reduce the size of drug particles, and greatly increase the solubility of poorly soluble drugs according to the Ostwald–Freundlich equation (Son et al., 2007). Therefore, we synthesized HM as a drug carrier for the treatment of SCI. However, due to the existence of a physiological barrier, HM has difficulty reaching the injury site, thus limiting the scavenging effect of HM on ROS. To overcome the above deficiencies, chitosan was chosen to modify HM to solve this problem. According to the literature reports, chitosan has a large number of positive charges, which can interact with the negatively charged plasma membrane on the brain capillary endothelium to cross the physiological barrier (Kravanja et al., 2019; Cortés et al., 2020). Therefore, HM can cross the physiological barrier with the help of chitosan modification. In addition, chitosan, as a natural polymer, has the advantages of biodegradability, biocompatibility, and modifiability (Li et al., 2018; Zhang et al., 2019). As a consequence, through modification of chitosan, CM has the function of crossing the blood–spinal cord barrier (BSCB) and eliminating ROS in the SCI environment, thereby reducing oxidative stress and alleviating neurotoxicity.

Resveratrol (Res), as a potential SCI therapeutic drug, exhibits a variety of pharmacological effects, such as good antioxidant, anti-inflammatory, neuroprotective, and antiapoptotic properties (Fu et al., 2018; Huang et al., 2019; Fan et al., 2021). However, due to its poor water solubility, low bioavailability, and difficulty in crossing physiological barriers to reach the injury site, its application in SCI is limited greatly (Zhang et al., 2014; Miguel et al., 2021). To solve these problems, CM was used as the delivery carrier of Res to improve its water solubility and bioavailability in this study. Moreover, the designed drug delivery system not only alleviated oxidative stress but also helped insoluble drug (Res) pass through the BSCB to decrease inflammatory and protect the neurons at the injured site. The therapeutic effect of Res for SCI was exerted to the maximum. *In vivo* and *in vitro* experiments, the oxidative stress reduction, anti-inflammatory, and neuroprotective effects of CMR were demonstrated after SCI, which should provide a new reference for the clinical treatment research of SCI.

## 2. Material and methods

### 2.1. Materials

Tetraethyl orthosilicate (TEOS), potassium permanganate (KMnO_4_), sodium carbonate (Na_2_CO_3_), ethanol, methanol, acetone, and paraformaldehyde were purchased from Sinopharm Holding Chemical Reagent Company, Ltd. (Shanghai, China). Dimethyl sulfoxide (DMSO), phloretin, carboxymethylcellulose sodium, and 3-(4,5-dimethylthiazole-2)-2,5-diphenyltetrazolium bromide (MTT) were obtained from Aladdin Chemical Reagent Co. ROS assay kit, malondialdehyde (MDA) content assay kit, superoxide dismutase (SOD) content assay kit, total glutathione (GSH-Px) content assay kit, and Hematoxylin-Eosin/HE staining kit were bought from Beyotime Biotechnology Co. Anti-HIF-1α, Anti-iNOS, anti-cox-2, anti-IL-1β, anti-IL-10, anti-bax, anti-bcl-2, anti-Cl caspase-3, anti-β-Tubulin, and anti-actin were purchased from Abcam. PC12 cells and BV2 cells were obtained from the cell bank of Chinese Academy of Sciences.

### 2.2. Synthesis of HM and CM

First, solid silica nanoparticles (SiO_2_) were prepared by the sol–gel method and used as templates (Bagchi et al., 2019). TEOS (15 ml) was dropped into a mixed solution of anhydrous ethanol (180 ml), water (65 ml), and ammonia (4.5 ml) under stirring at room temperature for 4 h. After centrifugation, the precipitate was dried at 45 °C to obtain SiO_2_ nanoparticles. Then, HM was prepared according to a previously described method (Zeng et al., 2020). Under the action of ultrasound, KMnO4 (300 mg) aqueous solution was added dropwise to the suspension of SiO_2_ nanoparticles (40 mg) under stirring, ultrasonicated for 30 min, and stirred at room temperature. The reaction lasted for 6 h. After centrifugation, the precipitate was obtained. The prepared MnO2-coated SiO_2_ was dispersed in 2 M Na_2_CO_3_ aqueous solution at 60 °C for 24 h to remove the SiO_2_ template. After centrifugation, the obtained product was defined as HM. HM (500 mg) was dispersed into 100 ml of chitosan solution (1 mg/ml), and the system was stirred for 4 h. After centrifugation, the obtained product was defined as CM.

### 2.3. Characterization of HM and CM

The morphology and structure of HM and CM were observed by transmission electron microscope (JEM-1200EX; JEOL, Tokyo, Japan). The zeta potential and particle size of the prepared HM and CM were measured with Zetasizer Nano ZS (Nano-ZS90, Malvern, UK). The current state of the drug was observed with differential scanning calorimeter ((DSC-60, Shimadzu, Kyoto, Japan)). The crystal structure was measured by X-ray diffraction (PXRD, Rigaku Denki, Japan).

### 2.4. Preparation of HMR and CMR

HMR and CMR were prepared by the adsorption method. Res (100 mg) was dissolved in acetone (1 ml). HM or CM (100 mg) was weighed and dispersed into an acetone solution of Res, stirred at room temperature for 6 h, centrifuged, and dried to obtain HMR and CMR. Then, the drug content was determined by UV–Vis spectrophotometry (UV-757CRT, Shanghai Precision Scientific Instrument Co., Ltd. Shanghai, China) at 306 nm, and the drug loading was calculated as follows:

Loadingcontent(LC)=(weightofloadeddrug)/(totalweightofnanocomposites)×100%

### 2.5. In vitro drug release study

Drug release was examined using a shaker (SHZ-82, Jintan Science Analysis Instrument Co., Ltd., Jiangsu, China). Phosphate buffered saline (PBS, pH 7.4 and 6.6) was used as a drug release medium. The samples (Res equivalent to 5 mg) were added to the dissolution medium (100 ml) and then shaken at 50 r/min in water bath with 37 °C. Next, the release solution (3 ml) was collected at a predetermined time interval for filtration. At the same time, the corresponding volume of PBS was supplemented to maintain a constant release medium volume. Finally, the concentration of Res was measured using UV (UV-757CRT, Shanghai Precision Scientific Instrument Co., Ltd. Shanghai, China) at a wavelength of 306 nm. This experiment was performed three times in parallel.

### 2.6. Hemolytic test

Hemolysis tests were used to evaluate *the in vivo* safety of HM and CM. PBS was used to wash fresh mouse blood until the supernatant was not red to obtain red blood cells. Red blood cells were then diluted to 2% (V: V) using PBS. After mixing different concentrations of HM and CM (50, 100, 200, 400, 800, and 1000 μg/ml) with 2% red blood cells, the mixtures were allowed to stand at room temperature for 3 h and centrifuged at 10,000 r/min for 3 min. Finally, the supernatant (100 μl) was taken into a 96-well plate and detected at 570 nm using a microplate reader (Versa Max, Molecular Devices, Sunnyvale, CA, USA). Water and PBS were used as positive and negative controls, respectively. Each sample was analyzed three times in parallel. The hemolysis percentage was calculated by the following formula:

Hemolysispercentage(%)=(Asample− A negativecontrol)/(Apositivecontrol−Anegativecontrol)

A hemolysis rate of more than 5% is regarded as hemolysis.

### 2.7. In vitro cell assay

#### 2.7.1. Cell culture

PC12 cells and BV2 cells were taken from the Cell Bank of the Chinese Academy of Sciences. The culture medium was DMEM (Gibco, Grand Island, NY, USA) containing fetal bovine serum (10%), penicillin (100 units/ml), and streptomycin (100 μg/ml) (Gibco, Grand Island, NY, USA). All cells were cultured at 37 °C in a humidified 5% CO_2_ atmosphere and digested with 0.25% trypsin during cell subculture. In addition, FBS supplemented with 10% DMSO was used to preserve cells at −80 °C.

#### 2.7.2. Construction of the oxygen and glucose deprivation (OGD) model

First, to simulate the environment of hypoxia and lack of sugar, the oxygen concentration in the three-gas incubator was adjusted to 1%, and then the medium was changed to Earl’s equilibrium salt solution without glucose. Afterward, PC12 cells and BV2 cells were treated with hypoxia and glucose deficiency for 3 h and then returned to normal culture for 24 h to use for further determination.

#### 2.7.3. Cell cytotoxicity in vitro

An MTT assay was conducted to evaluate the cytotoxicity of HM and CM on PC12 cells and BV2 cells. PC12 cells and BV2 cells were seeded into 96-well plates at a density of 5000 cells/well and allowed to adhere overnight. Then, different concentrations of HM and CM (0, 1, 5, 10, 20, 40, 60, 80, and 100 μg/ml) were added to the 96-well plates and incubated for 48 h at 37 °C. Next, MTT aqueous solution (5 mg/ml) was added to 96-well plates and cultured for 4 h in the dark. After that, the supernatant was removed from the 96-well plates, and DMSO (150 μl) was added to each well. Finally, the absorbance values (OD) were measured at 492 nm using a microplate reader (Versa Max, Molecular Devices, Sunnyvale, CA, USA) after 10 min of shock at room temperature. The cell survival rate was calculated with the following formula:

cellviability(%)=ODt/ODc×100%

ODt represents the absorbance of the treated cells, and ODc represents the absorbance of the control cells.

#### 2.7.4. Cellular uptake in vitro

CM and HM were labeled with fluorescein isothiocyanate (FITC). First, HM (500 mg) was added to an ethanol solution containing 4% APTES. Then, the suspension was placed in a round-bottomed flask and stirred in reflux at 77 °C for 10 h. After vacuum drying, the obtained product was defined as NHM. Subsequently, NHM (50 mg) was dispersed in 1 mg/mL FITC anhydrous ethanol solution and stirred for 3 h. After centrifugation, the precipitate was vacuum-dried to obtain FITC-labeled HM. Finally, chitosan was coated to obtain FITC-labeled CM.

FITC-labeled HM and FITC-labeled CM were used to observe cellular uptake. First, PC12 cells and BV2 cells were cultured in a confocal culture dish until reaching 80% confluence, and then incubated in DMEM containing HM-FITC and CM-FITC (40 μg/ml) for 3 h at 37 °C. After that, the cells were fixed with 4% paraformaldehyde for 30 min, permeabilized with 0.1% Triton for 15 min, and then incubated with goat serum for 2 h to block nonspecific staining. Next, the primary antibody (anti-β-Tubulin) was incubated overnight at 4 °C. The cells were washed with PBS three times and incubated with secondary antibody (FITC-labeled goat, anti-rabbit IgG) for 2 h. Then, after the cells were washed with PBS, the nuclei were stained with DAPI. Finally, the cellular uptake was observed and imaged by confocal laser scanning microscopy (CLSM, BioTek Instruments, Winooski, VT, USA).

### 2.8. In vivo experiment

#### 2.8.1. Animals and surgical procedures

All animal protocols were approved by the Experimental Animal Ethics Committee of Jinzhou Medical College for Scientific Research. Ten to twelve-week-old C57BL/6J (1/2 male, 18–25 g) mice were used for the experiment and raised in the SPF experimental Animal Center of Jinzhou Medical College. Allen’s method was utilized to establish the model of SCI. In brief, after mice were anesthetized with pentobarbital sodium, the back hair was removed, and povidone iodine was used to disinfect the back. Then, a T9/T10 laminectomy was performed to expose the thoracic spinal cord. Next, a modified impactor (2 mm diameter, 12.5 g, and 2 cm in height) was used to strike the exposed dorsal surface of the spinal cord. Mice in the sham group were subjected to laminectomy without contusion. Mice with SCI were then randomly divided into five groups including Sham group, SCI group, CM group, Res group, and CMR group. CM, Res, and CMR were dispersed in saline solution (5 mg/kg) and injected by the caudal vein (injection volume 100 μl). Among them, the mice of CM group, Res group, and CMR group were given after waking up 1 h. The frequency of administration was once a day and lasted for 7 days. The sham group and SCI group were injected with physiological saline. Penicillin was administered daily to prevent infection, and manual urination was performed twice daily until bladder function was restored.

#### 2.8.2. In vivo imaging

FITC-labeled CM and HM were injected into SCI mice via the tail vein. The heart, liver, spleen, lung, kidney, brain, and spinal cord were removed at different time points, and the fluorescence signals were detected using an *in vivo* imaging system (IVIS Spectrum, PerkinElmer) at excitation wavelengths of 518 and 494 nm.

#### 2.8.3. Western blot

Proteins were extracted from the spinal cord tissue of each group (1 cm around the injury site) by RIPA lysis buffer. The protein concentration of each group was quantified with a BCA protein kit. After equal amounts of samples in each group were added to a sodium dodecyl sulfate polyacrylamide gel, SDS-PAGE was utilized to separate the proteins. Then, the proteins were transferred to polyvinylidene fluoride (PVDF) membranes. Subsequently, the membranes were blocked with 5% skim milk powder for 2 h and incubated with primary antibodies at 4 °C overnight. The primary antibodies and dilution ratios were as follows: anti-bcl-2 (1:1000), anti-bax (1:1000), anti-Cl caspase-3 (1:1000), anti-IL-6 (1:1000), anti-IL-1β (1:1000), and anti-cox-2 (1:1000). The next day, the secondary antibody was incubated at room temperature for 2 h. Finally, the signal was displayed by the Tanon 5500 gel imaging system (Tanon, Shanghai, China). ImageJ software was used to analyze the signal intensity.

#### 2.8.4. Pharmacokinetic study in SD rats

Pharmacokinetic (PK) studies were performed using Sprague–Dawley rats. The SD rats (200 ± 20 g) were purchased from the SPF Experimental Animal Center of Jinzhou Medical College. Twelve SD rats were randomly divided into three groups (*n* = 3). Res, HMR, and CMR (equivalent to 20 mg/kg) were injected into the rats through the tail vein. Blood was collected at different time points (5 min, 15 min, 30 min, 1 h, 2 h, 4 h, 6 h, 8 h, 12 h, and 24 h), and plasma was obtained by centrifugation at 3000 rpm. Phloretin solution (10 μl, 4 μg/ml) as an internal standard was added to rat plasma (200 μl), and then methanol solution (400 μl) was added to precipitate the protein. After swirling for 5 min, the samples were centrifuged for 10 min at 10,000 rpm. The obtained supernatant (20 μl) was analyzed by high-performance liquid chromatography (HPLC) (Shimadzu LC-2030). Acetonitrile and water (70:30) were used as the mobile phase, and the detection wavelength was 306 nm. The pharmacokinetic parameters were calculated using Pksolver version 2.0.

#### 2.8.5. Nissl staining

Paraffin sections of the obtained spinal cord tissues were routinely dewaxed and then stained with a Nissl kit. After dehydration in absolute ethanol for 3 min, the tissue was rinsed with xylene. Next, neutral gum was used to seal these sections. Finally, the number of Nissl-positive cells was observed under an FV10I confocal microscope (Olympus, Tokyo, Japan).

### 2.9. Oxidative stress experiments

PC12 cells (1*10^5^ per well) were seeded into 24-well plates and allowed to adhere overnight. After PC12 cells were treated with OGD for 3 h, CM, Res, and CMR were added to each well and cocultured for 24 h. Then, a ROS fluorescent probe (DCFH-DA) was added to the cells and incubated for 30 min in the incubator. Finally, the production of ROS by cells was immediately observed with a fluorescence microscope.

After PC12 cells were treated with different groups, the protein was extracted for detecting ROS levels, SOD activity, and content of GSH and MDA by kits (Beijing Beyotime).

Similarly, the protein of the spinal cord in different groups was extracted to detect SOD activity and content of GSH and MDA in tissues.

### 2.10. Immunofluorescence analysis

The spinal cords were placed in 10% sucrose solution, 20% sucrose solution, and 30% sucrose solution to remove water and cut into slices after OTC imbedding. Frozen sections of spinal cord were dried at room temperature and then washed three times with PBS. Next, 0.3% Triton X-100 was used to drill for 15 min. After washing three times with PBS, goat serum was employed to block for 2 h. Subsequently, the primary antibody (anti-NeuN, anti-HIF-1α, anti-Cl caspase-3, and anti-IL-1β) was incubated with the sections overnight at 4 °C. The next day, the appropriate secondary antibody was added and incubated for 2 h at room temperature. Finally, the sections were stained with DAPI for 15 min and filmed with a FV10I confocal microscope. The fluorescence intensity of each group was analyzed with ImageJ software.

Cell samples were fixed with 4% PFA for 30 min, and the rest of the experimental steps were carried out as described above. The used the primary antibodies were anti-β-tubulin, anti-iNOS, and anti-Cl caspase-3. Finally, the cells were imaged with a confocal laser scanning microscopy (CLSM, BioTek Instruments, Winooski, VT, USA).

### 2.11. Functional analysis

The Basso Mouse Scale (BMS) system and footprint analysis were used to assess the recovery of mice hind limb motor function after SCI. On Days 3, 7, 14, and 28 after SCI, the mice were placed in an open space and observed by two examiners blinded to the experimental design. After 5 min of observation, the motor scores were evaluated, and footprints were collected. The BMS score ranges from 0 to 9 points. Among them, 0 points indicates complete paralysis, and 9 points indicates normal exercise. In addition, the weight of the mice was recorded immediately after the behavioral assessment to preliminarily assess the recovery of the mice in the different treatment groups.

### 2.12. Statistical analysis

All data were presented as the mean ± standard deviation (SD), and statistical analysis was performed using GraphPad Prism (version 8.0). The data were analyzed by one-way analysis of variance (ANOVA) to compare the mean difference between groups. When the variance was not equal, the Kruskal–Wallis test was used. When the *P* value was less than 0.05, it was considered to be statistically significant.

## 3. Results and discussion

### 3.1. Preparation and characterization of HM and CM

**Scheme 1.**
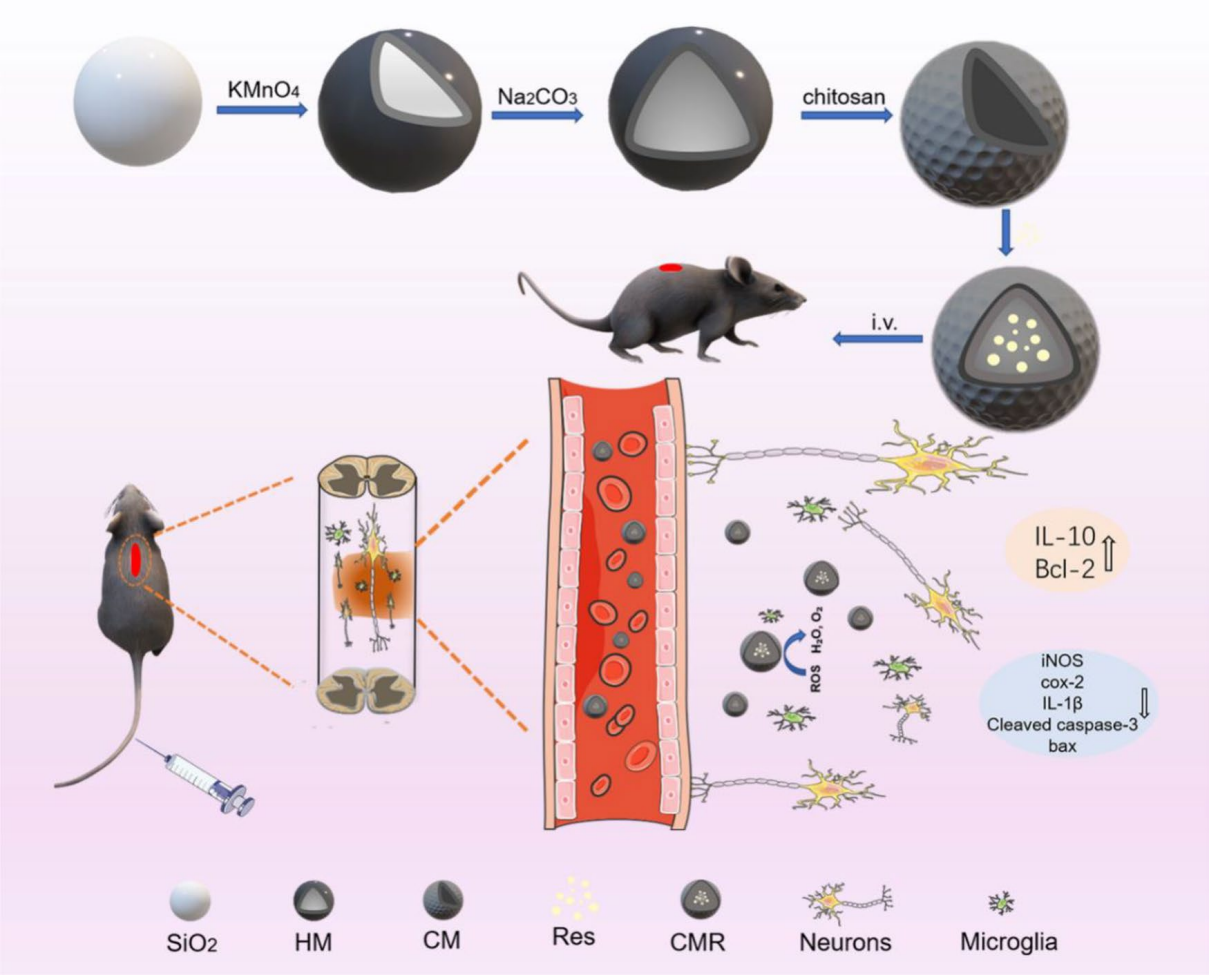
Scheme diagram of the CMR preparation and its action mechanism in SCI treatment.

The main preparation process of CMR and its mechanism for treating SCI are shown in Scheme 1. First, HM prepared by the hard template method was used as a drug carrier due to its high drug loading and ROS scavenging ability. Subsequently, chitosan was applied to modify HM, which could enable it to cross the BSCB. Res was embedded in CM by a physical adsorption process, and after intravenous injection, CMR could enter the central circulatory system via the BSCB and reach the injury site. CM and Res in CMR could synergistically relieve oxidative stress after SCI, and further reduce inflammation and protect neurons.

**Figure 1.**
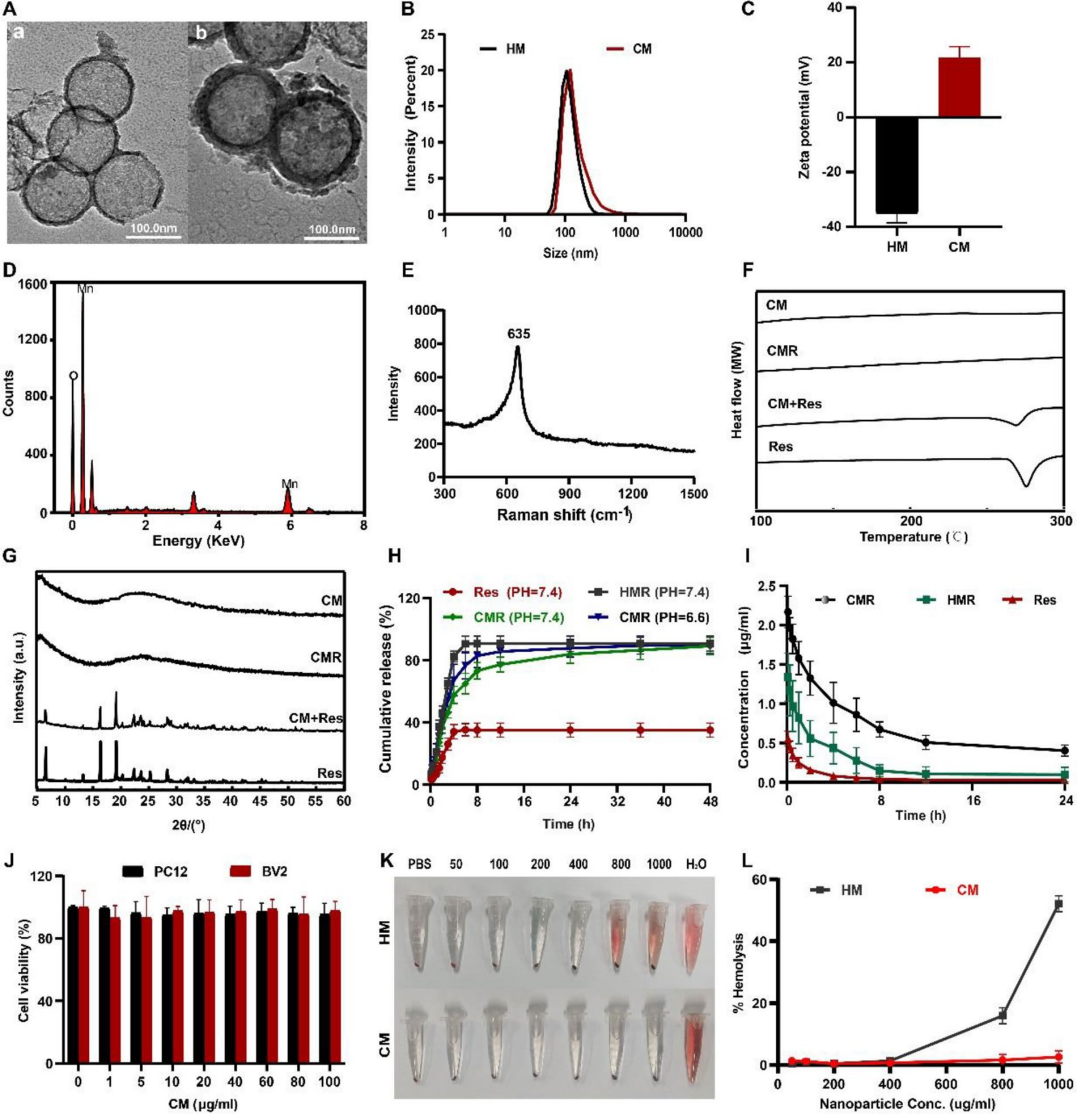
Characterization of HM and CM. (A) TEM imaging of HM (a) and CM (b). (B) The particle size of HM and CM. (C) The zeta potential of HM and CM. (D) The element spectrum of HM. (E) The characteristic peaks of the Raman spectrum of HM. (F) The DSC patterns of Res, CM + Res, CMR, and CM. (G) The PXRD patterns of Res, CM + Res, CMR, and CM. (H) *In vivo* drug release curves of Res, HMR, and CMR. (I) The plasma concentration-time curve of Res, HMR, and CMR. (J) The cytotoxicity of CM on PC12 cells and BV2 cells. (K, L) The hemolysis experiment diagram and hemolysis rate of HM and CM. All data represented the mean ± standard deviation (*n* = 3).

As shown in Figure 1(A), the structures of HM and CM were characterized by TEM. The TEM images clearly showed that HM and CM were monodisperse regular hollow spheres with the same inner diameter of approximately 110 nm. The outer diameter of HM was approximately 120 nm. After coating with chitosan, the outer diameter of the CM was slightly increased to approximately 130 nm. The particle size analysis results were consistent with the above TEM results. The particle size of HM was mainly distributed at 112.5 ± 12.35 nm, and that of CM was mainly distributed at 132.3 ± 13.28 nm (Figure 1(B)). The zeta potential of HM was −35.2 ± 2.35 mV, and the zeta potential of CM was 22.8 ± 1.23 mV. The positive zeta potential of CM after coating chitosan was because chitosan had many amino groups with a positive charge, which indicated that chitosan was successfully coated on the surface of HM (Figure 1(C)). Energy dispersive X-ray spectroscopy (EDS) was used to detect the composition of HM. The results showed that HM was made of O and Mn (Figure 1(D)). The results of Raman spectrum analysis further established the successful synthesis of HM and showed a characteristic band of MnO2 at 635 cm^−1^ (Figure 1(E)). Due to the inner hollow nanostructure of HM, the loading content of Res in CMR could reach 21.39 ± 2.53%, and the hollow nanostructure was favorable for reducing the size of drug particles and improving the dispersion state of the drug. DSC and PXRD analysis verified this view. The DSC profiles of Res, CMR, CM, and the physical mixture were shown in Figure 1(F). The melting peak of Res was 272 °C, indicating that Res was a crystalline drug. The DSC curves of CM and CMR were smooth, and no melting peak appeared, but the physical mixing of CM and Res (CM + Res) showed the melting peak of Res at 272 °C, indicating that Res in CMR was amorphous. PXRD was performed to further verify the above results. As shown in Figure 1(G), there were characteristic diffraction peaks of Res at 6.6°, 13.3°, 16.4°, 19.3°, 22.4°, 25.3°, and 28.3°, but no characteristic diffraction peaks of Res appeared in CM and CMR. At the same time, the characteristic diffraction peak of Res still existed in CM + Res. The results of PXRD also demonstrated that Res in CMR was amorphous, which helped to increase the water solubility of Res. In addition, after SCI, the injury site is an acidic lesion with ischemia and hypoxia (Woo et al., 2004; Liu et al., 2020; Xi et al., 2020). Studies have pointed out that MnO2 nanoparticles have good pH sensitivity (Liu et al., 2019; Rong et al., 2021). Based on pathological and material characteristics, the focal pH was simulated *in vitro* to characterize the responsive release function of the microenvironment. The drug release results were shown in Figure 1(H). The release rate of Res in the release medium PBS (pH = 7.4) was very low, and the release amount in 48 h was only 35.1 ± 4.50%, while the release amount of HMR in 8 h was 90.5 ± 4.94%. This was attributed to the amorphous dispersion state of Res in HMR. Compared with HMR, the release amount of CMR reached 85.6 ± 5.05% at 24 h, showing a good sustained-release effect. At the same time, the drug release results of CMR showed that the release amount at pH 6.6 (82.9 ± 7.49% at 8 h) was faster than that at pH 7.4 (73.2 ± 75.28% at 8 h). These results suggested that CM could degrade under acidic conditions and release Res more quickly. Furthermore, significant degradation of HM and CM after 8 h in PBS solution at pH 6.6 was observed by the TME, which further confirmed this result (Figure S1). This meant that CM could effectively regulate the release rate and release site (rapid release under acidity at the injury site) of Res, which contributed to increased bioavailability. The results of the pharmacokinetic experiment supported this point of view. As shown in Figure 1(I), the *t*_1/2_ values of free Res, HMR, and CMR were 0.991 ± 0.981 h, 2.19 ± 0.613 h, and 5.01 ± 0.698 h, respectively. The AUC_0-_*_t_* of Res, HMR, and CMR were 14.4 ± 2.66 μg/ml*h, 4.36 ± 1.99 μg/ml*h, and 0.857 ± 0.172 μg/ml*h, respectively. The AUC_0-_*_t_* of CMR was eightfold greater than that of Res and threefold greater than that of HMR. This indicated that CMR significantly improved the bioavailability of Res. The cytotoxicity test and hemolysis test were used to study the biological safety of CM. MTT results showed that compared with HM, the survival rate of PC12 cells and BV2 cells treated with CM were higher, both reaching more than 95%, indicating that CM can reduce cytotoxicity and has good biocompatibility (Figures S2 and 1(J)). Hemolysis test results showed that 800 μg/ml HM exhibited a hemolytic state, while 1000 μg/ml CM still did not cause hemolysis (Figure 1(K,L)). In addition, H&E staining of the collected heart, liver, spleen, lung, and kidney showed no significant difference between the control group and other treatment groups, indicating that CMR has good biological safety (Figure S3). The results of the cytotoxicity test hemolysis test and H&E staining showed that CM had good biosafety. Based on these conclusions, CM has potential as a central nervous system drug delivery vehicle.

**Figure 2.**
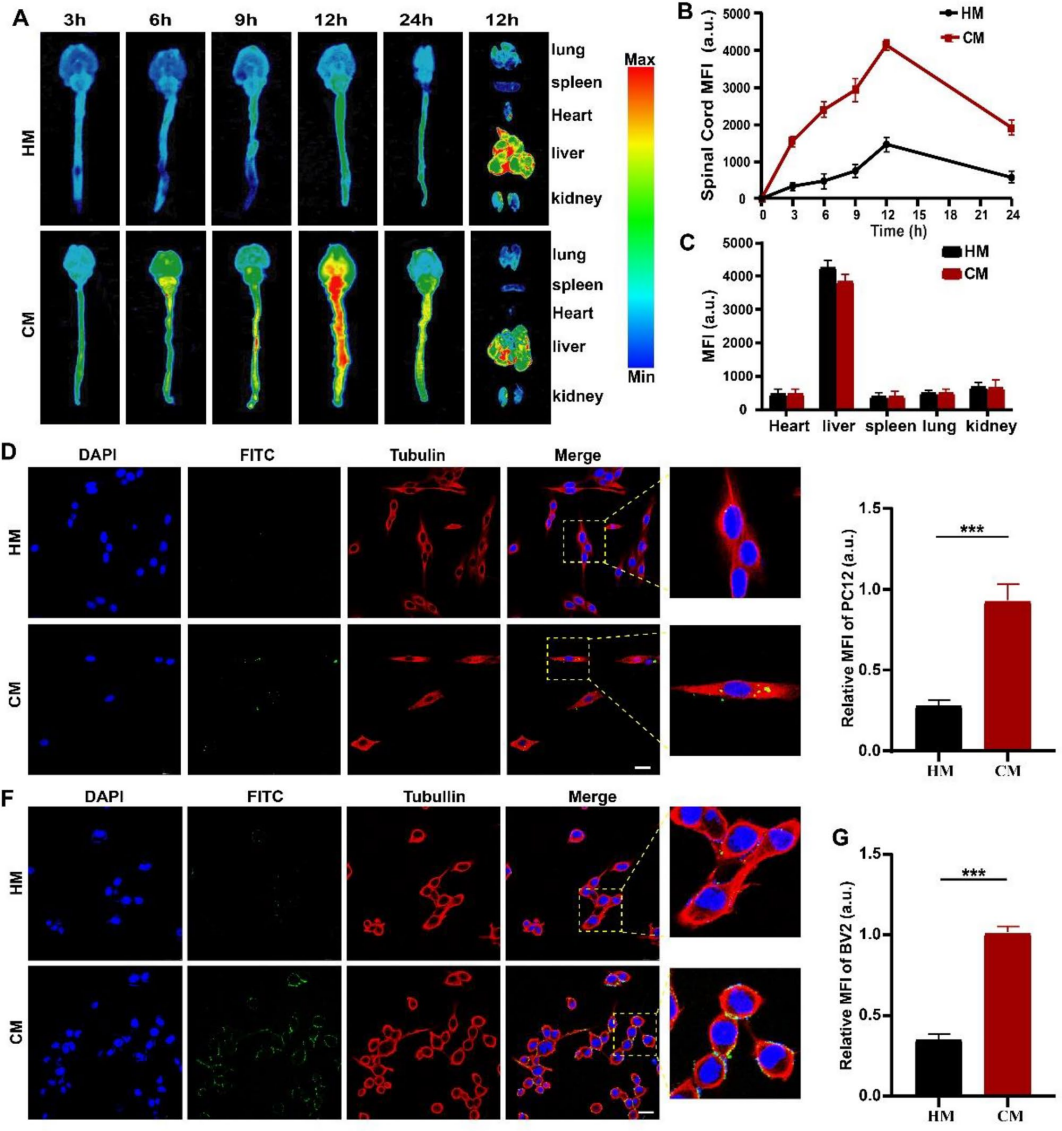
Cell uptake and the ability of CM to cross the BSCB. (A) The brain, spinal cord, and organ fluorescence imaging of SCI mice after tail vein injection of HM and CM at different time points, respectively. (B) Fluorescence quantitative analysis of HM and CM in spinal cord at different time points. (C) Fluorescence quantitative analysis of SCI mice organs (heart, liver, spleen, lungs, and kidneys) in HM group and CM group. (D) CLSM imaging and magnified images of PC12 cells treated with HM and CM at 4 h. HM and CM (green), nucleus (blue), and cell cytoskeleton (red). All images have a scale of 25 µm. (E) Fluorescence quantitative analysis of HM and CM (green) in PC12 cells. (F) CLSM imaging and magnified images of BV2 cells treated with HM and CM at 4 h. HM and CM (green), nucleus (blue), and cell cytoskeleton (red). All images have a scale of 50 µm. (G) Fluorescence quantitative analysis of HM and CM (green) in BV2 cells. All data represented the mean ± standard deviation (*n* = 3). *P < 0.5, **P < 0.01, ***P < 0.001.

**Figure 3.**
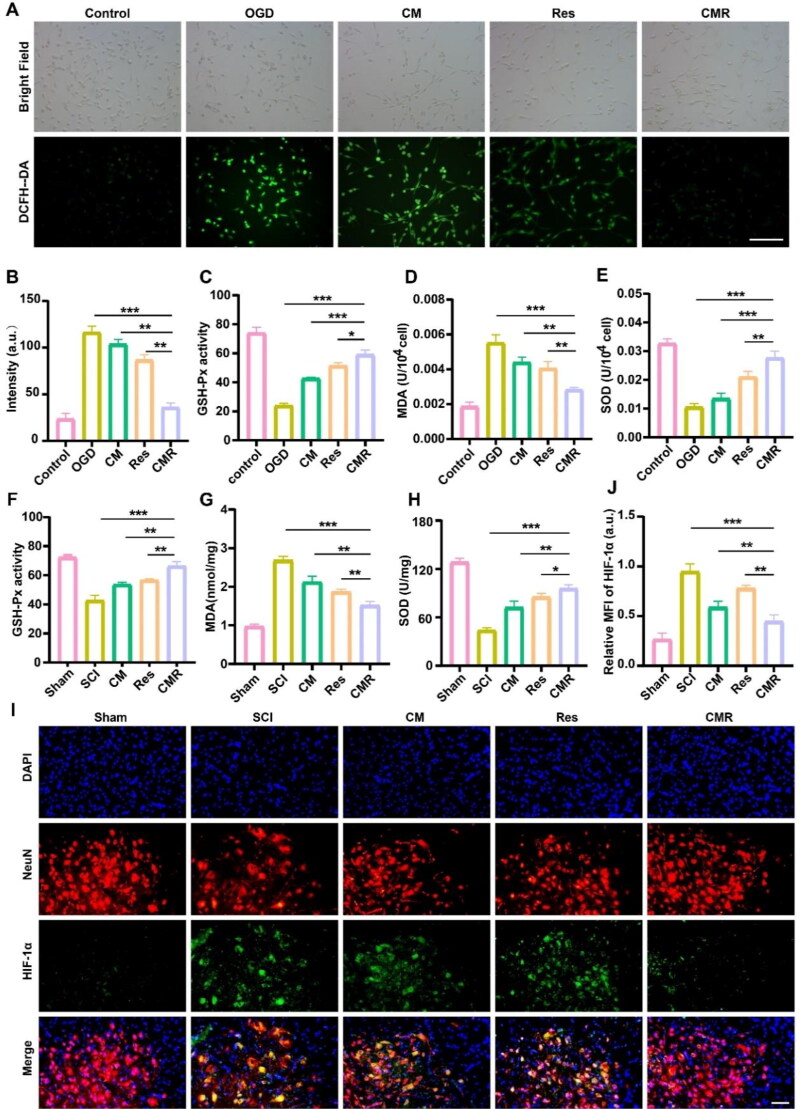
Antioxidant stress activity of CMR. (A) Fluorescence images in different groups of PC12 cells processed by DCFH-DA probe. The scale of all images is 50 µm. (B) ROS fluorescence quantitative analysis of different groups. (C–E) The concentration of GSH, MDA, and SOD in PC12 cells of different groups (*n* = 6). (F–H) The concentration of GSH, MDA, and SOD in spinal cord tissue of different groups (*n* = 6). (I) Fluorescence images show the expression of hypoxia-inducible factor in different groups of spinal cord tissues. Cell nuclei were stained with DAPI (blue), neurons were stained with Neun (red), and hypoxia-inducible factor were stained with HIF-1α (green). The scale bar of all images is 50 μm. (J) Quantitative analysis of HIF-1α fluorescence intensity in different group. All data represented mean ± standard deviation. *P < 0.05, **P < 0.01, ***P < 0.001.

### 3.2. Cell uptake and the ability of CM to cross the BSCB

*In vitro* cell and *in vivo* imaging experiments were conducted to test the CMR’s cellular uptake and the crossing the BSCB ability. The BSCB, as one of the unique barrier structures of the central nervous system, is mainly responsible for restricting and regulating the entry of extramedullary molecules into the spinal cord and maintaining spinal homeostasis (Bartanusz et al., 2011). Previous studies have shown that the positive charges provided by chitosan on the surface of nanoparticles can interact with the negatively charged plasma membrane on the capillary endothelium, facilitating the crossing of physiological barriers (Reshma et al., 2017; Cortés et al., 2020). *In vivo* imaging results showed that HM did not accumulate significantly in the brain and spinal cord of mice. Compared with the HM group, a significantly stronger fluorescence intensity was detected in the CM group, which reached the highest after 12 h (Figure 2(A,B)). The results fully demonstrated that CM could successfully cross the BSCB to reach the injury site. Moreover, we observed that the fluorescence intensity of the liver was stronger in the fluorescence results of the heart, liver, spleen, lung, and kidney of the HM group and the CM group, indicating that it was mainly metabolized by the liver (Figure 2(A,C)). In addition, CLSM was applied to monitor the cellular uptake of CM by PC12 cells and BV2 cells. In Figure 2(D–G), the uptake results of PC12 cells and BV2 cells showed that the cellular uptake of CM was significantly higher than that of HM. The uptake rates of HM by PC12 and BV2 were 30.4 ± 2.3% and 35.2 ± 5.1%, respectively. The uptake rates of CM by PC12 and BV2 cells were 75.7 ± 1.7% and 78.6 ± 3.6%, respectively. The results showed that CMR could be ingested by PC12 and BV2 cells at the cellular level. In conclusion, after CM crosses the BSCB, it can be taken up by neurons and microglia. Due to the pH sensitivity of CM, the drug can be rapidly released at the injury site to play a therapeutic role in the ischemia, hypoxia, and acidity microenvironment after injury.

### 3.3. Antioxidant stress activity of CMR

ROS produced under normal physiological conditions can participate in the redox reaction of normal cells, which is crucial for normal cell metabolism (Pani et al., 2000). After SCI, oxidative stress occurs at the injury site, resulting in a large amount of ROS production (Liu et al., 2004; Zrzavy et al., 2021). Recent studies have reported that oxidative stress is an important pathogenic factor of secondary injury (Taoka et al., 1995). Fluorescence imaging of DCFH-DA was performed *in vitro* to detect ROS in each group. The results showed that after OGD treatment, the fluorescence signal of the OGD group (116.3 ± 4.013 a.u.) was the strongest and that of the CM group (101.6 ± 3.726 a.u.) and Res group (86.7 ± 2.214 a.u.) decreased successively, among which the fluorescence signal of the CMR group (36.4 ± 3.327 a.u.) was the lowest, indicating that CMR had the best effect on alleviating oxidative stress (Figure 3(A,B)). Moreover, the literatures have pointed out that MDA, as an end product of oxidative stress-induced lipid peroxidation, increases after SCI, while the levels of glutathione peroxidase (GSH-PX) and superoxide dismutase (SOD), as antioxidant enzymes, decrease after SCI (Del Rio et al., 2005; Wang et al., 2016; Singh & Devasahayam, 2020). Therefore, GSH, MDA, and SOD were detected in this experiment. The GSH, MDA, and SOD results showed that after CMR treatment, the MDA content decreased significantly, and the GSH and SOD expression increased significantly (Figure 3(C–E)). These results indicated that CMR had a good inhibitory effect on oxidative stress. To further verify this conclusion, SCI animal models were selected for *in vivo* study. The experimental results were consistent with the *in vitro* results. In the CMR treatment group, MDA content was the lowest, and GSH and SOD content was the highest (Figure 3(F–H)). SCI can cause hypoxia at the injury site, thereby stimulating the overexpression of HIF-1α (a hypoxia-inducible factor) (Wang et al., 2017). Therefore, HIF-1α can reflect the degree of tissue hypoxia. The anterior horns of spinal cord were co-stained with Neun/HIF-1α to measure the expression of HIF-1α in Figure 3(I–J). The results showed that after CMR treatment, the relative fluorescence intensity of HIF-1α was the lowest (0.447 ± 0.0663 a.u.) compared to the CM group (0.590 ± 0.0611 a.u.), the Res group (0.783 ± 0.0306 a.u.), and SCI group (0.982 ± 0.0198 a.u.). This demonstrated that CMR could decompose ROS into water and oxygen by catalysis, alleviate the hypoxic environment, and reduce oxidative stress.

### 3.4. Anti-inflammatory activity of CMR

**Figure 4.**
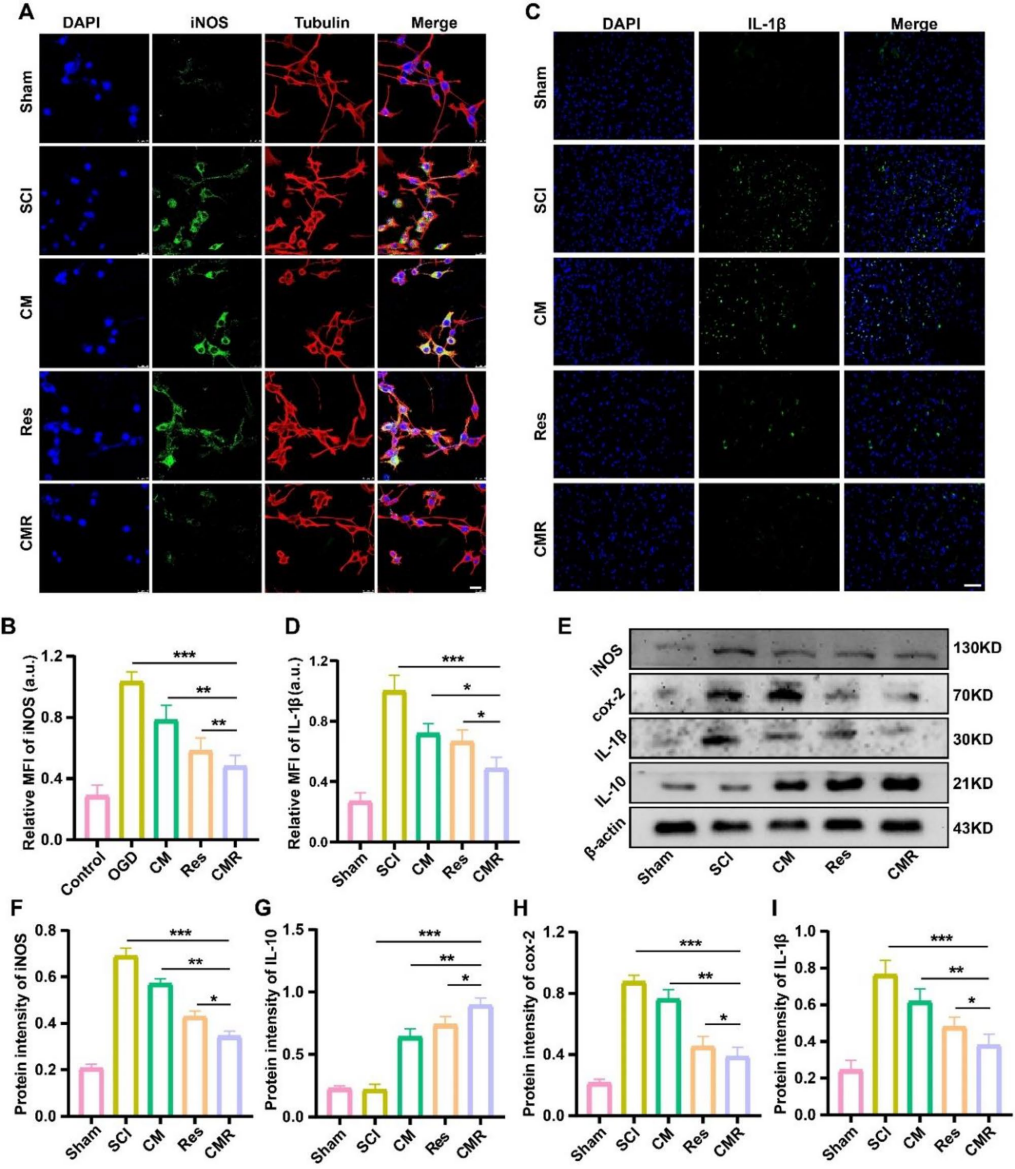
Anti-inflammatory activity of CM. (A) CLSM imaging of BV2 cells in each group. The nucleus was stained with DAPI (blue), the inflammation protein was stained with iNOS (green), and BV2 cells were stained with β-tubulin (red). The scale bar of all images is 25 μm. (B) Quantitative analysis of iNOS fluorescence intensity in each group. (C) Fluorescence microscope imaging of spinal cord tissue in each group. Cell nuclei were stained with DAPI (blue) and inflammation factors were stained with IL-1β (green). The scale bar of all images is 50 μm. (D) Quantitative analysis of IL-1β fluorescence intensity in each group. (E) Western blot was used to detect the expression of cytokines (iNOS, cox-2, IL-1β, and IL-10) in spinal cord tissue of each group. (F–I) Quantitative analysis of the expression levels of iNOS, cox-2, IL-1β, and IL-10. All data represent mean ± standard deviation (*n* = 6). *P < 0.05, **P < 0.01, ***P < 0.001.

Inflammation plays a major role in the pathological process of secondary injury, as does oxidative stress (Fan et al., 2020). After SCI, microglia are activated immediately, leading to an increase in proinflammatory cytokines such as iNOS, IL-1β, and cox-2 (Hellenbrand et al., 2021). Therefore, effective reduction of these proinflammatory factors will be conducive to the recovery of SCI. In this study, the anti-inflammatory effect of CMR was examined using CLSM to detect the expression of iNOS in different groups *in vitro*. The results showed that iNOS relative fluorescence intensity in the OGD group (1.03 ± 0.0458 a.u.) was the strongest after OGD induction, indicating that OGD caused BV2 cells to be in an inflammatory state. The relative fluorescence intensity of iNOS in the CM group (0.783 ± 0.0164 a.u.) and Res group (0.581 ± 0.0167 a.u.) decreased sequentially, and the iNOS relative fluorescence intensity of the CMR group (0.481 ± 0.0291 a.u.) was the lowest except for the Sham group, which was only 0.465 times that of the OGD group (Figure 4(A,B)). This indicated that the anti-inflammatory effect of CMR was the most significant. To further verify this result, *in vivo* experiments were carried out using immunofluorescence and western blot. The results showed that compared with the SCI group (1.03 ± 0.0540 a.u.), IL-1β expression in the CM group (0.783 ± 0.0471 a.u.), and Res group (0.581 ± 0.0171 a.u.) was decreased, while the expression of IL-1β in the CMR group (0.481 ± 0.0302 a.u.) was the most significantly decreased, which was 0.456 times that of the SCI group (Figure 4(C,D)). Furthermore, the western blot results showed that CMR significantly reduced the protein expression of iNOS, cox-2, and IL-1β compared with the SCI, CM, and Res groups, which were 0.501 times, 0.487 times, and 0.475 times that of the SCI group, respectively. The expression of IL-10 protein in the CMR, Res, and CM groups was 3.856 times, 3.282 times, and 2.826 times that of the SCI group, respectively (Figure 4(E–I)). Taken together, these results demonstrated that CMR could produce outstanding anti-inflammatory effects.

### 3.5. Anti-apoptotic activity of CMR

**Figure 5.**
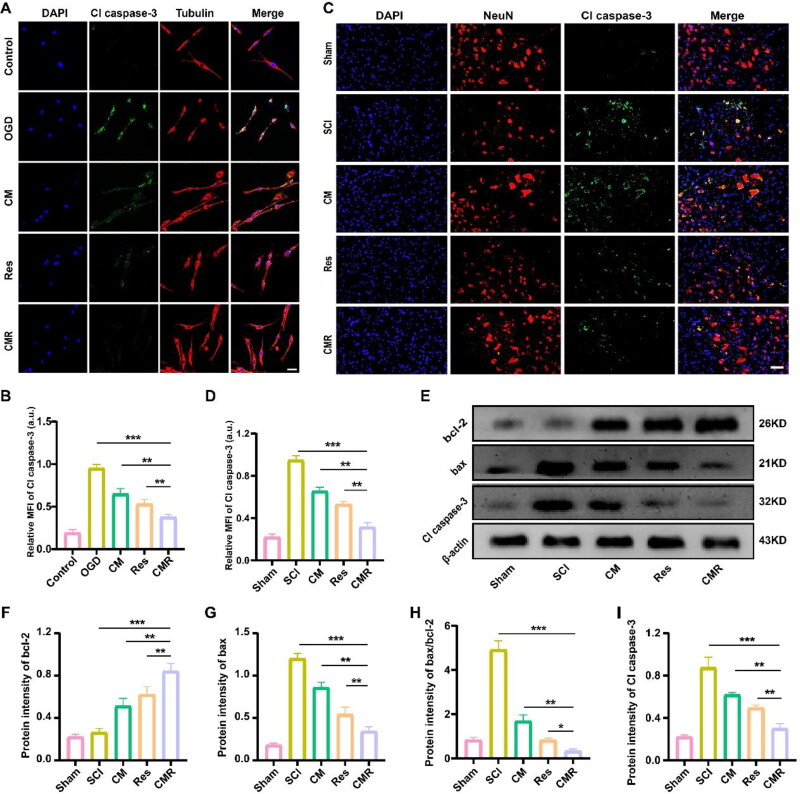
Anti-apoptotic activity of CMR. (A) CLSM imaging of PC12 cells in each group. The nucleus was stained with DAPI (blue), the apoptotic protein was stained with Cl caspase-3 (green), and PC12 cells were stained with β-tubulin (red). The scale bar of all images is 25 μm. (B) Quantitative analysis of Cl caspase-3 fluorescence intensity in each group. (C) Fluorescence microscope imaging of spinal cord tissue in each group. Cell nuclei were stained with DAPI (blue), neurons were stained with NeuN (red), and apoptotic factors were stained with Cl caspase-3 (green). The scale bar of all images is 50 μm. (D) Quantitative analysis of Cl caspase-3 fluorescence intensity in each group. (E) Western blot was used to detect the expression of cytokines (cl caspase-3, bcl-2, and bax) in spinal cord tissues of each group. (F–I) Quantitative analysis of the expression levels of bcl-2, bax, bax/bcl-2, and Cl caspase-3. All data represent mean ± standard deviation (*n* = 6). *P < 0.05, **P < 0.01, ***P < 0.001.

Both inflammation and oxidative stress induce neuronal apoptosis after SCI, which can affect the recovery of motor function in SCI sufferers (Wang et al., 2018, 2020). An *in vitro* immunofluorescence assay was used to verify the anti-apoptotic effect of CMR on neurons. In Figure 5(A,B), the results of immunofluorescence showed that Cl caspase-3 relative fluorescence intensity was the highest after OGD treatment (0.953 ± 0.0437 a.u.). The fluorescence intensity of CM group and Res group were 0.651 ± 0.0602 a.u. and 0.532 ± 0.0518 a.u., respectively, while fluorescence intensity of CMR group (0.377 ± 0.0299 a.u.) was the weakest. It was shown that CMR had the strongest anti-apoptotic ability compared with other groups. To further verify this result, *in vivo* immunofluorescence experiments were carried out. The anterior horns of spinal cord were co-stained with Neun/Cl caspase-3. As shown in Figure 5(C,D), Cl caspase-3 expression in the CMR group was 0.309 ± 0.0195 a.u., which was significantly lower than the 0.956 ± 0.0213 a.u., 0.659 ± 0.0307 a.u., and 0.528 ± 0.0110 a.u. in the SCI, CM, and Res groups. In addition, western blot was used to analyze the protein levels of apoptosis factors in each group (Figure 5(E)). The results showed that the protein levels of bax and Cl caspase-3 in the CMR group decreased more obviously than those in the SCI, CM, and Res groups (Figure 5(I,G)). In contrast, the protein expression of bcl-2 was the lowest in the SCI group and slightly increased in the CM and Res groups, and showed the most conspicuous increase after CMR treatment (Figure 5(F)). The ratio of bax/bcl-2 in the CMR group was significantly lower than that in the SCI, CM, and Res groups (Figure 5(H)). These results indicated that CMR had good anti-apoptosis ability, thereby playing a neuroprotective role.

### 3.6. The validity and safety activity of CMR

**Figure 6.**
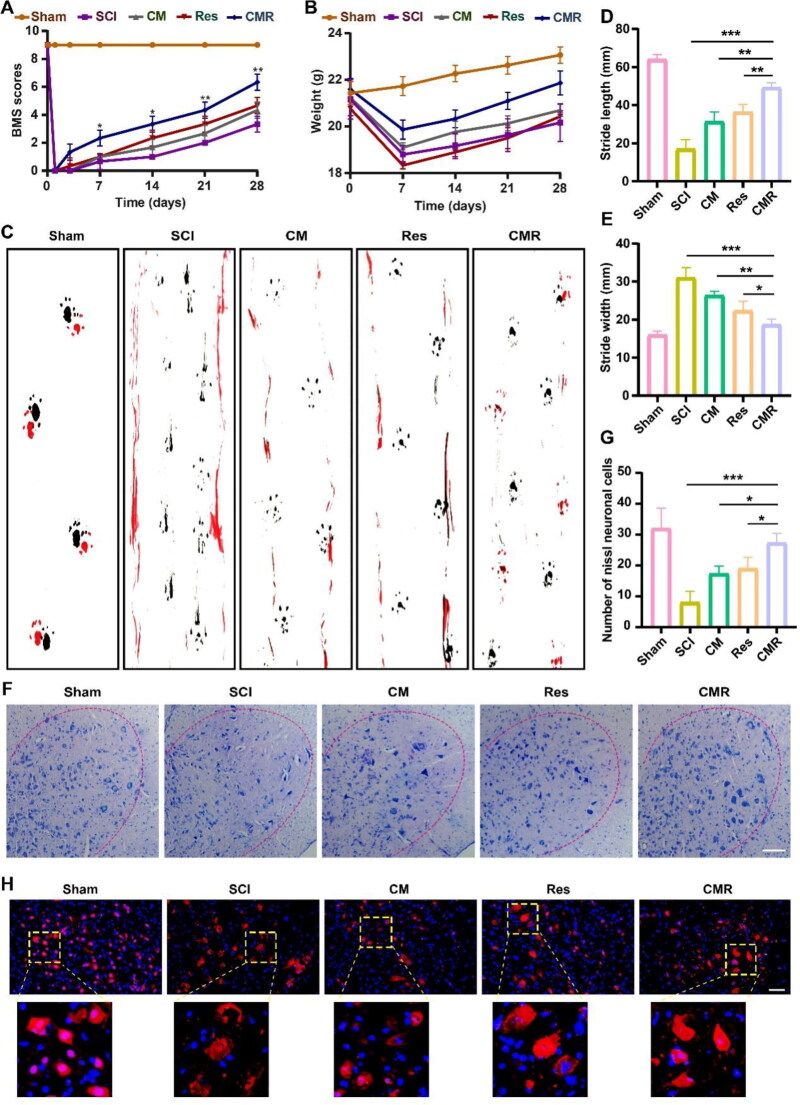
The validity and safety activity of CMR. (A) The BMS score was used to statistically analyze the recovery of motor function in different groups of SCI mice. (B) Statistical analysis of the body weight of mice in different groups within 28 days. (C) Footprint analysis of different groups at 28 days (black, forelimbs; red, hindlimbs). (D, E) Statistical analysis of step length and step width. (F) Nissl staining was observed for the number of ventral motor neurons in different groups for 7 days after SCI. All images have the same scale of 50 μm. (G) Statistics of the number of Nissl-positive neurons in different groups. (H) Immunofluorescence images magnification images of Neun-labeled neurons in different groups. All images had the same scale of 50 μm. All data represented the mean ± SD (*n* = 6). *P < 0.5, **P < 0.01, ***P < 0.001.

To further determine the effect of CMR treatment on the recovery of neurological function after SCI, behavioral tests, body weight analysis, and Nissl staining were performed. The BMS scores from 1 d to 28 d after SCI were shown in Figure 6(A). The score of the sham group was 9 points, and the score of the SCI group was 0 points. Compared with the CM group and Res group, the score of the CMR group was the highest, indicating that the SCI mice had better recovery of motor function after CMR treatment. In addition, in Figure 6(B), the body weight of all mice decreased significantly after SCI. After 28 days, the CMR group recovered most significantly compared with the other groups. This demonstrated that CMR was the most effective treatment for SCI. This could also be observed from gait. On Day 28, footprint experiment results showed that the front paws and hind paws of the sham group basically overlapped with the longest stride length, the narrowest step width and good coordination. The front and hind paws of the mice in the SCI group did not overlap, the hind limbs could not support their own weight, and they had the shortest and the widest strides. Compared with the CM group and the Res group, the mice of the CMR group had significant hind limb recovery with longer stride length and narrower step width (Figure 6(C–E)). It showed that CMR treatment enhanced the motor coordination of SCI mice, confirming the efficacy of CMR in promoting the recovery of SCI. Furthermore, Nissl staining was also conducted to count the number of neurons to further observe the effect of CMR on motor function recovery. The results showed that the number of neurons in the CMR group was higher than that in the SCI group, CM group, and Res group, thus confirming the good neuroprotective effect of CMR (Figure 6(F,G)). In addition, neuron fluorescence results showed that compared with SCI group, CM group, and Res group, the morphology of neurons after CMR treatment was more complete. This result further confirmed CMR had good effectiveness (Figure 6(H)). In summary, CM can be used as a novel drug delivery vehicle for SCI treatment and maybe also have potential for widely using in other neurological diseases.

## 4. Conclusion

In summary, this study successfully synthesized a nanoplatform of chitosan-modified hollow manganese dioxide nanoparticles to deliver Res for the treatment of SCI. As the carrier of Res, CM had high drug loading and presented sustained release. *In vitro* cell and *in vivo* animal experiments showed that CM could effectively transport Res to the injury site, inhibit oxidative stress, the inflammatory response, and neuronal apoptosis, and promote motor function recovery in SCI mice. Therefore, CMR can provide a new therapeutic strategy for the clinical treatment research of SCI.

## Supplementary Material

Supplemental Material

## Data Availability

The raw data required to reproduce these findings cannot be shared at this time as the data also forms part of an ongoing study. The processed data (used in this manuscript) required to reproduce these findings can be shared at this time through personal request.
