# Supplementary material for: Chitosan-modified hollow manganese dioxide nanoparticles loaded with resveratrol for the treatment of spinal cord injury
Source: Drug Deliv. 2022 Jul 28;29(1):2498–512. doi: 10.1080/10717544.2022.2104957 (PMC9477490; doi:10.1080/10717544.2022.2104957)
Supplement: Supplemental Material [file IDRD_A_2104957_SM8877.docx]

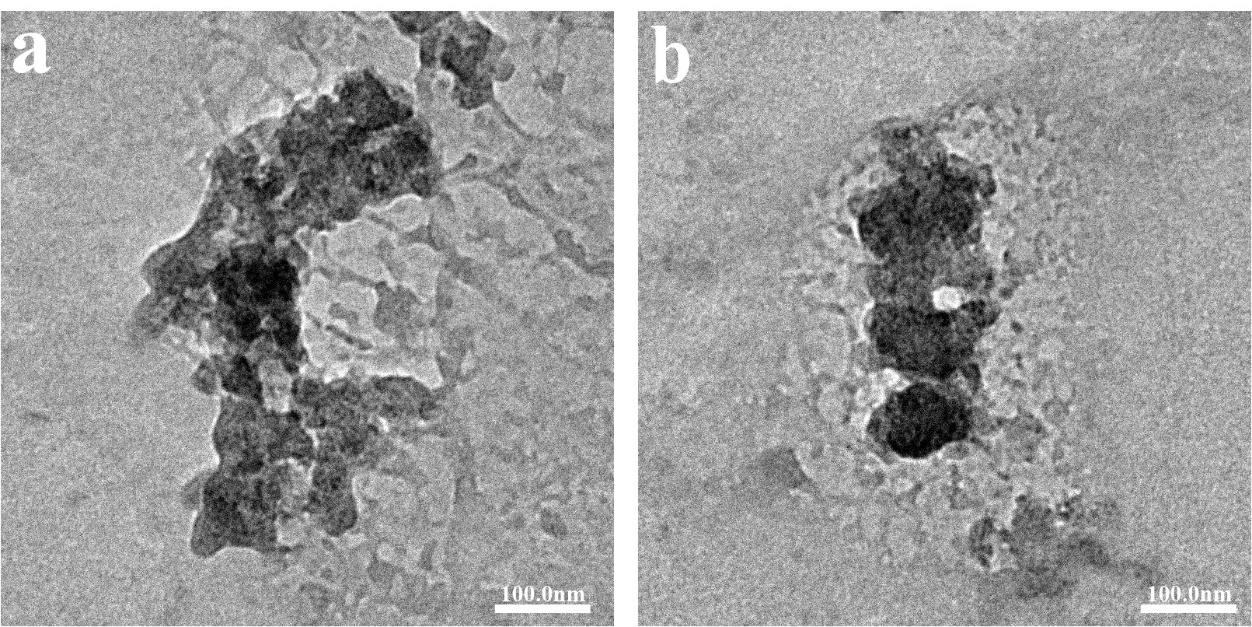


**Fig. S1.** **TEM imaging of HM (a) and CM (b) were processed in pH 6.6 medium for 8 h.**

*
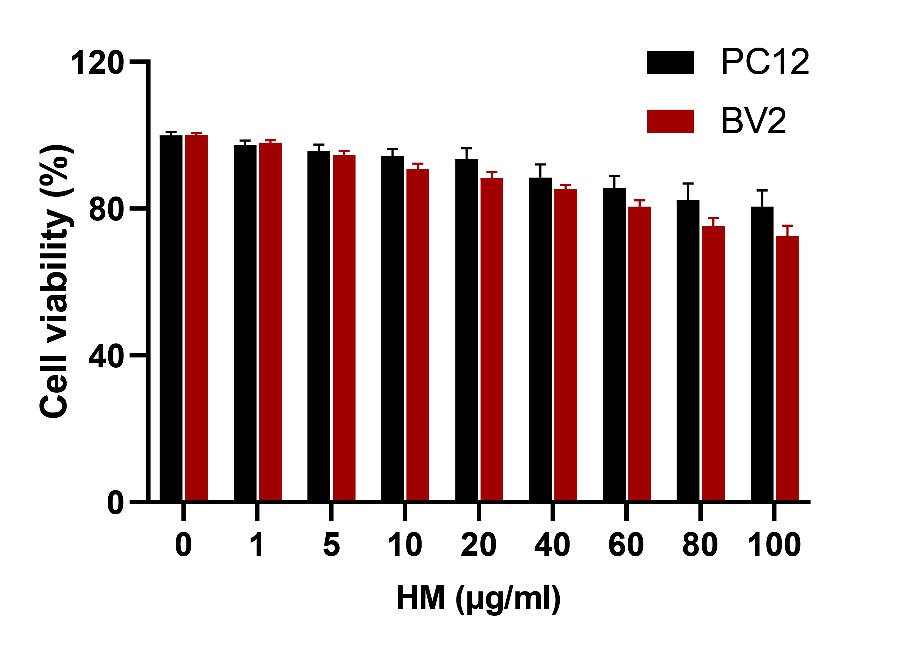
*

**Fig. S2. The cytotoxicity of HM on PC12 cells and BV2 cells.78**

***
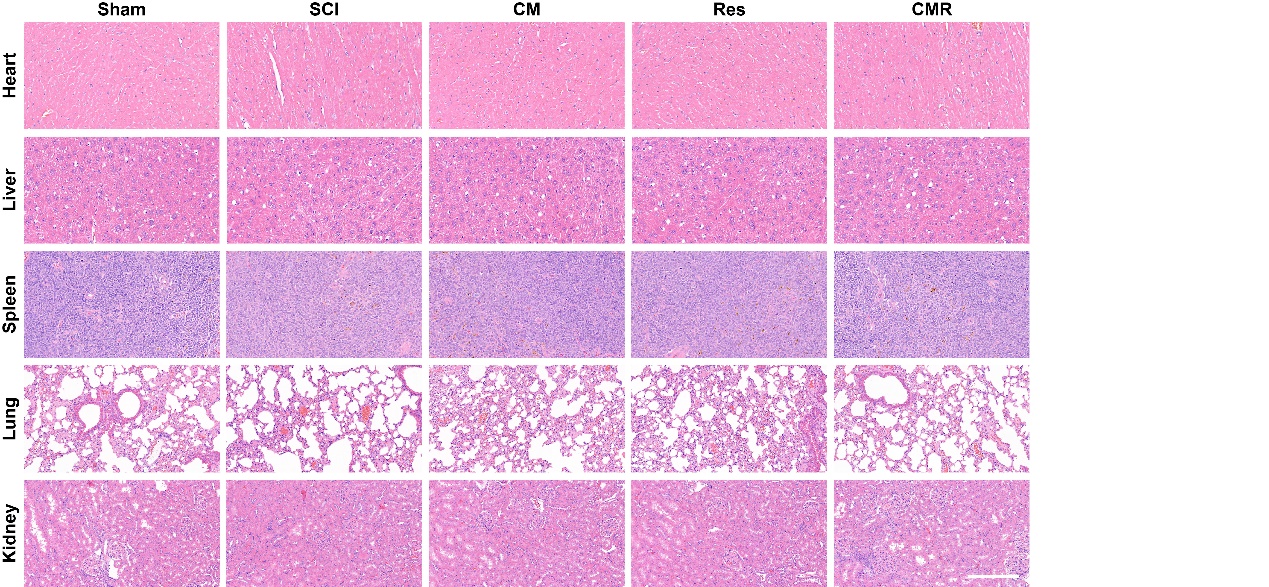
***

**Fig. S3 H&E staining was performed on tissue sections of heart, liver, spleen, lung and kidney from different groups**
